# Levo-transposition of the Great Arteries in an Adult Patient: Management Considerations and Treatment Strategy

**DOI:** 10.7759/cureus.4306

**Published:** 2019-03-23

**Authors:** Matthew R Lozier, Alexandra M Sanchez, John J Lee

**Affiliations:** 1 Internal Medicine, University of Miami, Holy Cross Hospital, Fort Lauderdale, USA; 2 Cardiology, Columbia University, Mount Sinai Medical Center, Miami Beach, USA

**Keywords:** levo-transposition of the great arteries, systemic right ventricle, double switch operation

## Abstract

Levo-transposition of the great arteries (L-TGA) is a rare congenital heart anomaly associated with the increased risk of developing heart failure (HF) as well as complete heart block at a young age. Due to limited data regarding the treatment strategy in the adult L-TGA sub-population, shared medical decision-making should occur between the patient and a team of physicians. Clinical status, age, and associated cardiac lesions or rhythm disturbances can affect patient outcomes. These factors should be considered prior to pursuing a surgical versus a medicinal approach.

## Introduction

Levo-transposition of the great arteries (L-TGA) is a rare congenital anomaly leading to progressive dysfunction of the systemic ventricle, which is a morphologic right ventricle (RV). These patients are at increased risk of heart failure (HF) as well as complete heart block at a younger age, and early preventative efforts should be made when possible [1]. We present a 37-year-old adult male with L-TGA and review management considerations as well as treatment strategies.

## Case presentation

**Figure 1:**
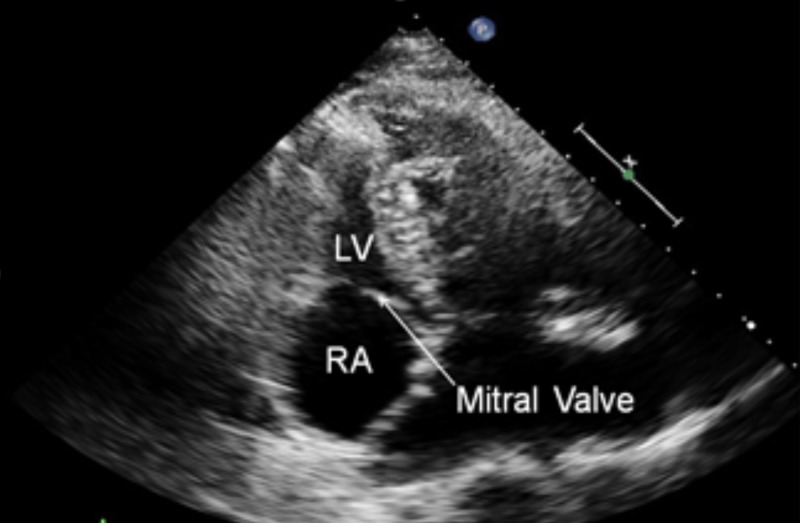
Transthoracic Echocardiogram Demonstrating atrial to ventricular discordance with the right atrium separated from the left ventricle by the mitral valve.

**Figure 2:**
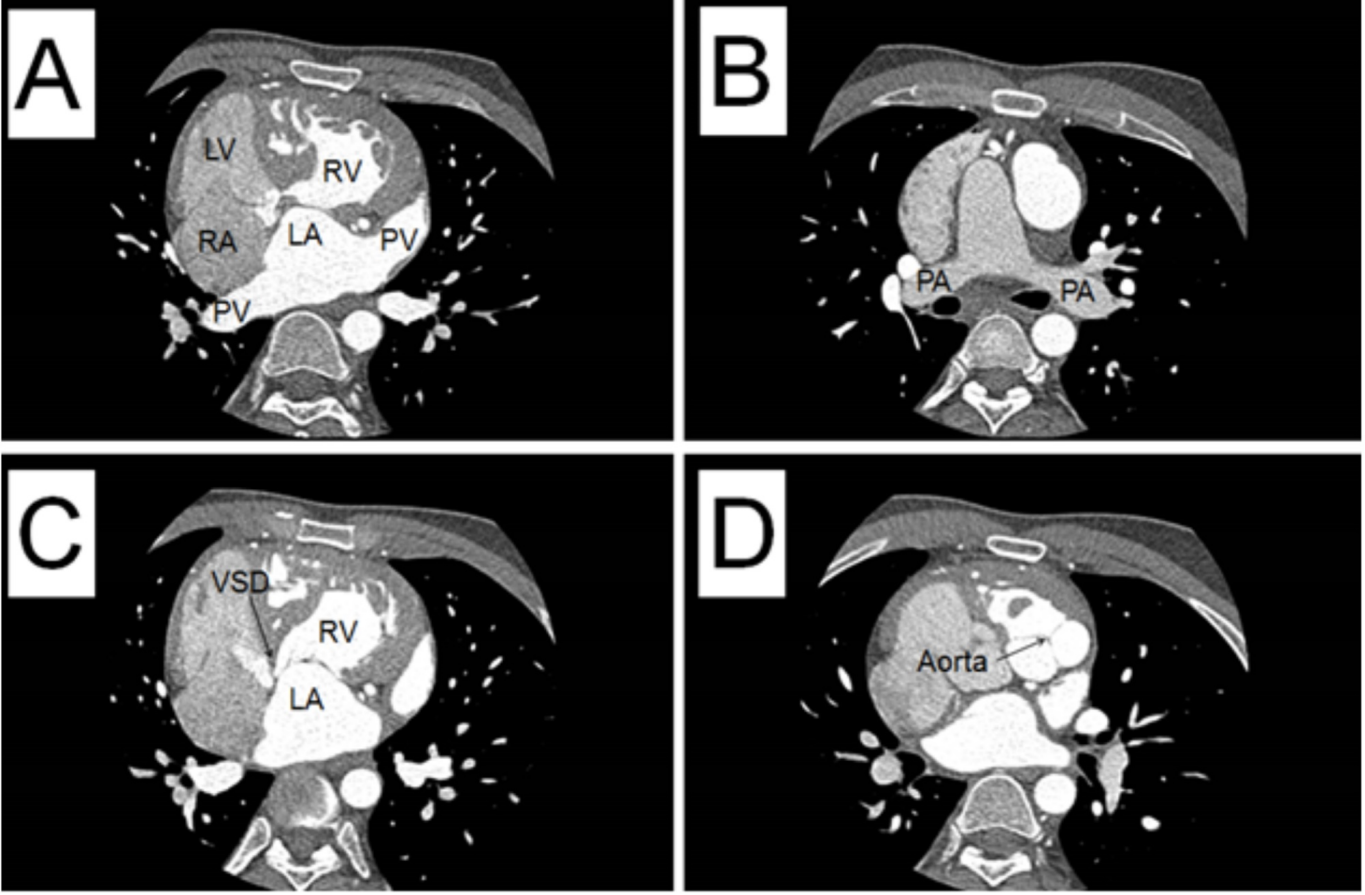
Cardiac Computed Tomography Angiography Demonstrating the sagittal view of A. The right atrium (RA) connected to the left ventricle (LV). Contrast in the pulmonary vein (PV) filling the left atrium (LA) to the right ventricle (RV). B. The pulmonary arteries (PA). C. Contrast from the LA to the RV with a ventricular septal defect (VSD) noted. D. Contrast in the aortic valve, which is projecting to the side.

A 37-year-old asymptomatic adult male with previously diagnosed L-TGA presented to the clinic to establish care. He reported normal development without cyanosis or functional limitation. On physical exam, III/VI holosystolic murmur and S4 were appreciated. The electrocardiogram demonstrated left axis deviation and Q waves in the early right precordial leads. Transthoracic echocardiogram demonstrated atrial to ventricular (AV) and ventricular to arterial (VA) discordance consistent with corrected transposition of the great arteries, as seen in Figure 1. There was a restrictive perimembranous ventricular septal defect and moderate right ventricular dilation with decreased systemic right ventricular function. Following these findings, cardiac computed tomography angiography was performed to further assess the congenital anomaly, as seen in Figure 2.

## Discussion

L-TGA patients typically present with nonspecific HF symptoms at an early age due to the morphologic RV functioning as the systemic pump. Although there is increasing favor for performing a double switch operation (correcting AV and VA discordance, allowing the left ventricle to function as the systemic pump) in infants and young children with L-TGA, older teens and adults have not fared as well with this procedure [1-3]. Medical management is, therefore, the more widely accepted treatment strategy. For those patients who have significant systemic AV or VA valve insufficiency, prompt surgical intervention is required [4]. Unfortunately, from the medicinal perspective, there is a paucity of data on how best to treat patients with L-TGA after a certain age. Due to this limitation, therapies are often based on the 2017 updated American Heart Association guidelines for HF (Stage C) with some caveats.

The goal of treatment is to reduce afterload and slow remodeling in order to improve or mitigate RV dysfunction, as the RV is not built to sustain the role of the left systemic ventricle in the long term. The principle behind this strategy is to decrease the pressure on the wall of the RV during ejection, similar to the medical approach in HF. Two such agents that will reduce afterload and may slow the remodeling process include angiotensin-converting enzyme inhibitors and angiotensin receptor blockers (ACE-I/ARBs) [5-7]. In addition, β-blocker use in patients with systemic right ventricles demonstrated an increase in EF and smaller RV end-diastolic area at the four-month follow-up, implying reduced cardiac remodeling [8-9]. However, it is important to note that L-TGA patients have an associated risk of developing complete heart block, so these HF agents are used with caution unless an in-situ pacemaker is present [9]. While diuretics are used for symptomatic relief and use is based on the treating institutions' preference, digoxin and aldosterone antagonists have not shown clinical benefits [10-12]. After a detailed discussion with our patient, he was initiated on an ACE-I and β-blocker with plans for up-titration as tolerated. It must be noted that while the data above demonstrate some improvement in function of the systemic RV with regards to the use of β-blockers and ACE-I/ARBs, there is no proven mortality benefit noted.

## Conclusions

Due to limited data in the adult L-TGA sub-population, medical management options are not well-supported by evidence. Therefore, each case should be tailored to the individual being treated. Factors such as clinical status, age, and associated cardiac lesions or rhythm disturbances should be reviewed by specialists prior to shared medical decision-making with the patient.
